# Oceanography and life history predict contrasting genetic population structure in two Antarctic fish species

**DOI:** 10.1111/eva.12259

**Published:** 2015-04-16

**Authors:** Emma F Young, Mark Belchier, Lorenz Hauser, Gavin J Horsburgh, Michael P Meredith, Eugene J Murphy, Sonia Pascoal, Jennifer Rock, Niklas Tysklind, Gary R Carvalho

**Affiliations:** 1 British Antarctic SurveyCambridge, UK; 2 School of Aquatic and Fishery Sciences, University of WashingtonSeattle, WA, USA; 3 NERC Biomolecular Analysis Facility, Department of Animal and Plant Sciences, University of SheffieldSheffield, UK; 4 School of Biological Sciences, Bangor UniversityBangor, Gwynedd, UK; 5 Present address: Department of Zoology, University of OtagoDunedin, 9054, New Zealand; 6 Present address: Campus AgronomiqueBP 709-97387, Kourou Cedex, France

**Keywords:** *Champsocephalus gunnari*, connectivity, Individual-based Modelling, *Notothenia rossii*, ocean circulation, planktonic dispersal, population genetics, Scotia Sea

## Abstract

Understanding the key drivers of population connectivity in the marine environment is essential for the effective management of natural resources. Although several different approaches to evaluating connectivity have been used, they are rarely integrated quantitatively. Here, we use a ‘seascape genetics’ approach, by combining oceanographic modelling and microsatellite analyses, to understand the dominant influences on the population genetic structure of two Antarctic fishes with contrasting life histories, *Champsocephalus gunnari* and *Notothenia rossii*. The close accord between the model projections and empirical genetic structure demonstrated that passive dispersal during the planktonic early life stages is the dominant influence on patterns and extent of genetic structuring in both species. The shorter planktonic phase of *C. gunnari* restricts direct transport of larvae between distant populations, leading to stronger regional differentiation. By contrast, geographic distance did not affect differentiation in *N. rossii*, whose longer larval period promotes long-distance dispersal. Interannual variability in oceanographic flows strongly influenced the projected genetic structure, suggesting that shifts in circulation patterns due to climate change are likely to impact future genetic connectivity and opportunities for local adaptation, resilience and recovery from perturbations. Further development of realistic climate models is required to fully assess such potential impacts.

## Introduction

Marine organisms with planktonic early life stages have traditionally been assumed to have high dispersal and weak population genetic structure. However, there is growing recognition of the role of oceanographic features in structuring populations, with frequent uncoupling of dispersal potential, gene flow and genetic differentiation (Selkoe et al. 2008). Features such as ocean fronts can restrict the exchange of planktonic larvae between proximate sites, leading to higher genetic differentiation over small spatial scales than might be expected from isolation-by-distance analyses (White et al. 2010; Schunter et al. 2011). Thus, the expectation of increasing genetic differentiation with geographic distance, or with decreasing planktonic phase, may be inappropriate for many species and geographic regions. Moreover, the relationship between dispersal and gene flow depends ultimately on successful reproduction of migrants in recipient populations and is not necessarily a function of immigrant larva recruitment. Developing an understanding of the key environmental factors and life-history stages influencing population genetic structure and connectivity are vital for effective conservation management, and for predicting the potential impacts of future climate change. Such information is especially pertinent in Antarctic waters that are experiencing unprecedented rates of oceanic warming (Meredith and King 2005; Whitehouse et al. 2008) and where localized collapses of exploited commercial fishes indicate high vulnerability to marked shifts in trophic relations (Kock 1992).

Because of the variety of factors affecting genetic differentiation among wild populations (Hauser and Carvalho 2008), empirical population genetic data cannot reveal the underlying processes that create and maintain patterns of genetic differentiation. In particular, the inference of population connectivity from genetic data alone can be misleading, primarily because of the impacts of variance in effective population size and nonequilibrium conditions on estimates of the fixation index, *F*_ST_ (Waples and Gaggiotti 2006; Faurby and Barber 2012). On the other hand, tracking dispersal in the marine environment is a challenge due to the microscopic size of eggs and larvae, their low concentration in the open ocean and the often considerable oceanic distances under consideration. Consequently, numerical modelling has become established as a key tool for the investigation of the dominant influences on larval transport and connectivity (e.g. review by Gallego et al. 2007). Such models simulate the probability of larval dispersal between populations, which can be used to generate matrices of connectivity and which underpin the ‘seascape genetics’ approach (Galindo et al. 2006). Simulated connectivity matrices are used to generate predictions of spatial genetic structuring for comparison with observed patterns. By combining realistic transport modelling with empirical estimates of the levels and distribution of genetic diversity, important insights into the drivers shaping population genetic structure can be gained. Importantly, recent advances in modelling (e.g. Galindo et al. 2010; Foster et al. 2012) now allow direct comparison of genetic and dispersal estimates through the incorporation of salient biological and physical characteristics. However, such comparisons are often qualitative, ignore small-scale spatial heterogeneity in water circulation or fail to incorporate a representative temporal range to capture intra- and interannual oceanographic variability, thereby complicating quantitative predictions of dispersal under changing environmental scenarios.

**Figure 1:**
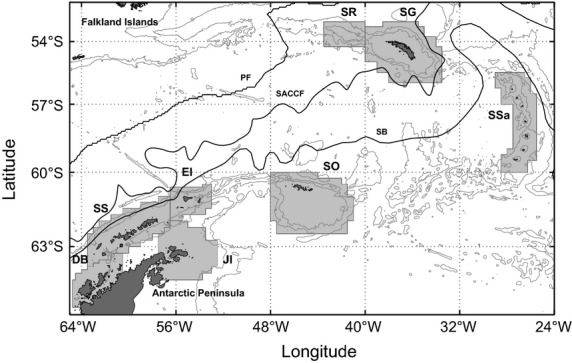
Map of the Scotia Sea region. Population boxes used in the connectivity analyses are shaded in light grey: DB (Dallman Bay), SS (South Shetland Islands), JI (Joinville Island), EI (Elephant Island), SO (South Orkney Islands), SR (Shag Rocks), SG (South Georgia), SSa (South Sandwich Islands). Solid black lines indicate the mean positions of the Polar Front (PF) following Moore et al. (1997), the Southern ACC Front (SACCF) and the southern boundary of the ACC (SB), both from Thorpe (2001). Bathymetry (in grey) is from Smith and Sandwell (1997, contours at 1000, 2500, 5000 and 7500 m).

We focus on fish populations within the Scotia Sea, Southern Ocean (Fig.1), because of the relative stability of the system and the clearly defined and relatively isolated shelf habitats of many Antarctic species. The horizontal flow in this region is dominated by the Antarctic Circumpolar Current (ACC), which is known to have been broadly stable since the last glacial maximum (McCave et al. 2014), with only small levels of shorter-period variability superposed (e.g. Meredith et al. 2004). This long-term stability is a great advantage compared to more commonly studied temperate systems, because populations are likely to have been separated for tens of thousands of generations and thus are more likely to approximate migration–drift equilibria. While there is some evidence that changes in shelf habitat availability may have caused population expansions in some Antarctic fishes (Damerau et al. 2014), regional patterns of genetic differentiation therefore theoretically derive predominantly from factors influencing connectivity rather than from historical factors such as recent separation or founder events (Marko and Hart 2011). Despite relative constancy of the physical environment in the past, the Scotia Sea and its immediate environs have recently experienced rapid increases in ocean temperature (Meredith and King 2005; Whitehouse et al. 2008), making it a prime location for considering the response of gene flow and population resilience to climate change.

Within the general stability of the ecosystem, there is considerable small-scale variation in oceanography. The ACC has a banded structure consisting of several fast-moving current jets separated by relatively quiescent zones of water (Orsi et al. 1995). Each of these jets is associated with a frontal region characterized by marked meridional gradients in temperature and/or salinity. While characterizations of the ACC have tended to depict these fronts as continuous circumpolar features, it is nonetheless well established that they are strongly spatially and temporally variable and that they split and merge frequently, with sometimes many more than three frontal features being instantaneously detectable (e.g. Sokolov and Rintoul 2009). Within the Scotia Sea, the ACC flows in a predominantly north-eastward direction, but is also characterized by strong mesoscale activity, whereby instabilities in the current cores lead to regular generation of eddies, with spatial scales up to tens of kilometres. This process, and to a lesser extent the ACC flow itself, has been shown to be sensitive to changes in wind forcing associated with coupled modes of climate variability (Meredith et al. 2004; Meredith and Hogg 2006), which gives the potential for both temporal and spatial variations in planktonic egg and larval dispersal.

Here, we use a seascape genetics approach, combining numerical modelling and microsatellite markers, to assess the impacts of early life history and oceanographic variability on marine connectivity and population genetic structure of two species of Antarctic fish, *Champsocephalus gunnari* and *Notothenia rossii*. These species share similar distributions but differ in aspects of their early life history that are expected to have a strong influence on their dispersal capabilities. Adult fish are restricted to shelf regions free of permanent ice and are not found in the deep pelagic waters beyond the shelf break (Kock 2005). Thus, genetic connectivity between isolated island populations is predicted to occur predominantly during the planktonic early life stages, which differ considerably between the two species. These species are therefore ideal for the application of seascape genetics to the investigation of the physical and biological influences on population genetic structuring.

*Champsocephalus gunnari* is widely distributed around sub-Antarctic islands and at the northern end of the Antarctic Peninsula (e.g. Gon and Heemstra 1990; Kock and Everson 2003) (Fig.1). They spawn in deep (100–350 m) inshore waters and fjords (Frolkina 2002; Kock 2005) and lay benthic adhesive eggs (Everson et al. 2001). Hatching at Shag Rocks occurs from May to July, and in two hatching periods at South Georgia, the first during May and then a later period between August and October (North and Murray 1992). At more southerly locations, egg incubation period is thought to increase to more than 6 months (Kock 2005), and based on the observed spawning times (Kock and Kellermann 1991; Kock et al. 2000), hatching in December and January may be inferred. The larvae spend approximately 3 months dispersing passively with the ocean currents (Duhamel 1995), vertically distributed within the upper 50 m of the water column (North and Murray 1992), before developing into juveniles. At this stage, they are able to undergo considerable diurnal vertical migration and can no longer be considered planktonic. Juveniles are found at greatest densities in specific areas of shelf (Frolkina 2002) governed primarily by the availability of key prey species such as copepods and mysids (Kock and Everson 1997). For the first 2 years of life, *C. gunnari* use the shallow shelf areas as a nursery ground (Duhamel 1995). The generation time of *C. gunnari* is 2–3 years (Gon and Heemstra 1990).

*Notothenia rossii* shares a similar distribution to *C. gunnari* but has a longer generation time of about 4–6 years (Gon and Heemstra 1990), and a much longer planktonic life-history stage. *Notothenia rossii* is believed to spawn in offshore shelf areas (Kock and Kellermann 1991) with depths of around 200–360 m (Kock et al. 2004). Unlike *C. gunnari*, it releases planktonic eggs, spawning between April and June in the northern Scotia Sea (Gon and Heemstra 1990; Kock and Kellermann 1991), and a few weeks later in the southern Scotia Arc (Kock and Kellermann 1991; Kock et al. 2000). The eggs develop into larvae after approximately 4 months in the Scotia Sea (Kock and Kellermann 1991), and then into blue fingerlings after about a further 3 months, at which stage they migrate inshore to kelp beds (Gon and Heemstra 1990). *Notothenia rossii* eggs are found in the top 100 m of the water column and their larvae in the upper 50 m (A. W. North*,* personal communication). Theoretically, therefore, it might be expected that *N. rossii*, with a planktonic duration approximately twice that of *C. gunnari*, experiences a higher degree of dispersal and greater genetic connectivity.

Our current understanding of gene flow in *C. gunnari* and *N. rossii* is generally poor and often contradictory. It is thought that *C. gunnari* forms separate populations on each of the island shelves in the Scotia Sea (Fig.1) although the status of the population around the South Sandwich Islands and the degree of connectivity between populations at Shag Rocks and South Georgia remain unclear (Kock 2005). Differences in reproductive characteristics (North 2005), parasite infestation (Sosinski and Janusz 2000) and allozyme analyses (Carvalho and Lloyd-Evans 1990) imply separate stocks at South Georgia and Shag Rocks. However, subsequent genetic analyses have shown low differentiation between these sites (Kuhn and Gaffney 2006; Young et al. 2012). Recent work on *C. gunnari* from the Atlantic sector of the Southern Ocean (Damerau et al. 2014) indicated marked genetic structuring between Bouvet Island, far to the east, and Scotia Sea samples and weak structuring between the more proximate Southern Scotia Sea populations at Elephant Island and South Orkney Islands. While Damerau et al. (2014) focused on a similar region to the present study, they considered transport to occur for a pelagic period of 400 days and, by analysis of drifter trajectories, concluded that transport during the pelagic phase was not a good estimator of gene flow. By contrast, in this study, we focus on transport over much shorter timescales corresponding to the early planktonic phases. Over larger geographic scales, data from previous genetic studies (e.g. Kuhn and Gaffney 2006) have shown that populations of *C. gunnari* in the Scotia Sea are strongly differentiated from populations in the Indian Ocean, indicative of very low connectivity at the circumpolar scale.

There are very few published data describing genetic differentiation in *N. rossii*. Allozyme analyses of *N. rossii* populations in the Indian Ocean found no significant differentiation between populations (Duhamel et al. 1995). Recent mtDNA analyses (J. Rock, unpublished data) showed only weak differentiation between Scotia Sea and Indian Ocean populations. Preliminary modelling studies (E. F. Young, unpublished data) suggested the potential for sporadic stepping-stone connectivity eastward between populations in the Scotia Sea and the Indian Ocean, via Bouvet Island. However, no connectivity was predicted between populations in the Indian and Pacific Oceans or between the Pacific Ocean and Antarctic Peninsula. These results suggest that circumpolar connectivity of *N. rossii* is unlikely and the probability of significant larval influx to the Scotia Sea from outside the region is low.

Here, we quantify the influences of oceanographic flow and early life-history variability on the population genetic structure of *C. gunnari* and *N. rossii* in the Scotia Sea. Patterns and drivers of connectivity between populations during the planktonic early life stages are described using numerical modelling techniques. Using a genetic projection model, the expected population genetic structures arising from predicted connectivity patterns are simulated and compared quantitatively with the empirical data from microsatellite analyses. Finally, the implications of the results for our understanding of gene flow in *C. gunnari* and *N. rossii* in the Scotia Sea are discussed in the context of climate change and population resilience.

## Methodology

### Ocean circulation model

Five-day mean velocity fields from a state-of-the-art oceanographic modelling framework Nucleus for European Modelling of the Ocean (NEMO) were provided by the National Oceanography Centre, Southampton, for the period 1996–2001. This period includes years with relative extremes in atmospheric forcing over the Southern Ocean, associated with extreme phases of large-scale coupled modes of interannual climate variability (see Meredith et al. 2008, for full discussion). Thus, our choice of years allows robust inferences concerning larval dispersal while accounting for possible extreme events in their physical forcing. The application of NEMO used for this study is global, with an eddy-permitting nominal horizontal resolution of 1/4° on a Mercator transformation, and a partial-step z-coordinate with 64 levels in the vertical. The model uses a bulk formulation for atmospheric forcing and has been run for the period 1978–2007. Full details may be found at http://www.nemo-ocean.eu/About-NEMO. NEMO has been widely used over a range of spatial scales and resolutions and has been shown to provide a good representation of the dominant oceanography of the Antarctic Peninsula and Scotia Sea region (Renner et al. 2009, 2012).

### Individual-based models

Mean flows from the circulation model were used to advect Lagrangian particles representing the early life stages of *C. gunnari* and *N. rossii*. The Lagrangian model has been applied previously to studies of zooplankton on and around the South Georgia shelf (Ward et al. 2007; Young et al. 2014) and has been adapted for the simulation of *C. gunnari* and *N. rossii* eggs and larvae (Young et al. 2012). In summary, particles were advected at each model time step (5 min) according to the imposed three-dimensional velocity field, using a second-order Runge-Kutta method. Additional horizontal and vertical diffusions were included using a random-walk approach (Dyke 2001), assuming isotropic diffusion, with horizontal and vertical diffusion coefficients of 10 and 0.0001 m^2^ s^−1^, respectively. These values fall within the range of established values from the published literature and are in accordance with observed values from the Southern Ocean (Sheen et al. 2013; Watson et al. 2013).

Within the model, particles representing the early life stages of the two fish species were released at the locations of known spawning populations described in published literature (population boxes; Fig.1) (Barrera-Oro and Casaux 1992; Parkes 2000; Everson et al. 2001; Frolkina 2002; Kock and Everson 2007; Kock et al. 2004). Population boxes were designed to encompass the shelf region at each location, with appropriate spawning areas within each population box identified by a comparison of local model depth with the spawning depth range for each fish species, with particles released in a random distribution within appropriate grid cells. One thousand particles were released per day at each site for the duration of the spawning periods, with species-specific characteristics assigned to each particle. Thus, for *C. gunnari*, model ‘larvae’ were released at hatching from each spawning site, distributed over the observed hatching periods, and dispersal was simulated for a planktonic phase of 3 months. *Notothenia rossii* eggs were released over the observed spawning period, with dispersal of eggs simulated for 4 months, and subsequent larval dispersal simulated for 3 months. There are no data to suggest that *N. rossii* larvae undergo diel vertical migration, and only weak evidence for this behaviour in late *C. gunnari* larvae (North and Murray 1992). Hence, vertical migration was not included, and model eggs and larvae were allowed to move randomly between the observed depth ranges described earlier.

Over a 4-week period centred on the end of the planktonic phase, the position of each model larva was compared with the population boxes associated with each known spawning population (Fig.1). If a larva was within one of these boxes at any point during the 4-week period, it was considered to have the potential to successfully recruit to a nursery ground at this site. Due to the coarse resolution of the source flow fields, with relatively poor representation of shelf regions, it was not appropriate to include more rigorous recruitment criteria here. The percentage of larvae from each spawning site successfully reaching each population box was calculated, and the results were combined into a single connectivity matrix ***M*** for each fish species describing the proportion of individuals arriving in a destination population (rows) from a given source population (columns). To account for egg and larval mortality, the connectivity matrices were multiplied by fixed daily mortality rates of 0.036 and 0.048 d^−1^ for eggs (*z*_e_) and larvae (*z*_l_), respectively, integrated over the planktonic periods. There are no data on egg and larval mortality rates for *C. gunnari* or *N. rossii*; these mortality rates were inferred from the temperature dependencies described by Pepin (1991), specifically

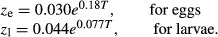
1 and assuming a temperature (*T*) of 1°C, representative of annual mean near-surface sea temperatures across the Scotia Sea (Mapping and Geographic Information, British Antarctic Survey, personal communication). The sensitivity of the genetic projection modelling to the imposed mortality rate is considered in Appendix A. The simulations were repeated for each of the five study years (1996–2000) to assess the effect of interannual variability in the underlying flow fields on predicted connectivity.

### Genetic projection model

The connectivity matrices described in the previous section are useful indicators of potential connectivity between discrete fish populations. However, to understand the implications of patterns of connectivity for regional population genetic structure, a methodology to project the connectivity forward in time is required. Here, we follow the approach described by Kool et al. (2010, 2011) whereby the connectivity matrix ***M*** is applied to a state matrix ***Q***_*t*_ containing information on the frequency of alleles of each type in each population at time *t*, to yield the expected state of the population at time *t *+* *1. This method allows for a sedentary adult phase, with offspring transported according to connectivity matrix ***M*** and subsequently merged back into the adult population. Thus, the projection equation is

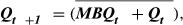
2 with ***B*** a diagonal matrix of birth rates for each population, and the top bar indicating row normalization. Using the assumption that individuals are uniquely labelled according to their population of origin by initially fixing populations for different alleles, ***Q***_**0**_ = ***K***, with ***K*** a diagonal matrix of carrying capacity values for each population. The connectivity matrix ***M*** was randomly chosen at each time step from the 5 year-specific matrices. Data on population sizes of the fish species at each spawning site are sparse; thus, the carrying capacities of each site were assumed to be the same. Mean birth rates were derived from empirical fecundity data (Kock and Kellermann 1991), reduced by 50% to allow for a 1:1 sex ratio and a further 20% to account for spawning by only a proportion (∼80%) of the total population each year. The resultant birth rates were 2800 larvae per individual *C. gunnari* and 20 000 larvae per individual *N. rossii*. The sensitivity of the genetic projection modelling to the imposed birth rate is considered in Appendix A.

Expected genetic connectivity was projected forward in time to generate estimates of allele frequencies for each population. Pairwise heterozygosity standardized fixation indices, 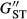, were calculated at each time step using equations from Meirmans and Hedrick (2011) for visualization of the projected genetic structure. Heterozygosity standardized 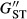 was used to allow comparison of differentiation between the simulated data sets with 7 or 8 alleles and the empirical microsatellite data with up to 46 alleles per locus. The projection model was halted once the maximum projected 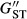 reached the maximum observed value. By considering all population pairs, we did not introduce bias in the model towards the projection of the observed population genetic structure, and this approach avoided the choice of an arbitrary number of projection time steps. Using such methodology allowed consideration of the relative connectivity among populations once the observed level of genetic structure was reached, though without allowing robust inference about the absolute level of differentiation.

### Model caveats

A degree of uncertainty in the genetics model projections arises due to the underlying assumptions and uncertainties in each of the three models. The oceanographic model is relatively coarse and unable to accurately resolve small-scale transport features such as cross-shelf exchange. However, the model has a realistic representation of larger-scale oceanographic features that are key for transport between regions of the Scotia Sea (see e.g. Renner et al. 2012), and the use of population boxes in the analysis of the Lagrangian model reduces the impact of the model resolution. Uncertainties in the Lagrangian and genetics projection models stem from the limited availability of information on key biological processes required for parameterization, in particular birth and mortality rates. In addition, the genetics projection model does not include mutation or age-related mortality, all of which will limit the ability of the model to project absolute 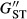 values. However, the sensitivity analyses (Appendix A) suggest that genetics projections are most strongly influenced by variability in the connectivity matrices arising from dispersal of the planktonic phases, and are relatively insensitive to a realistic range of birth and mortality rates. Thus, we expect the relative structure projected by the model to be similar to that observed if migration of the early life stages is a dominant influence on the genetic structure.

As with most empirically derived estimates of genetic differentiation, there is an implicit assumption that populations are at equilibrium between gene flow and genetic drift (Whitlock and McCauley 1999) when in reality, such conditions are rarely met. As the model does not include mutation, it cannot reach a classical mutation–migration–drift equilibrium. Instead, the model will always converge on a single allele in all populations (unless populations are completely isolated) and so we stopped the model when the empirical 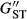 was reached. However, the somewhat unrealistic approach is indeed a strength in relation to our objective here. In the initial state of the model, each population was fixed for a different allele, in complete contrast to likely biological patterns, where genetic differentiation is initially small and increases over time as populations diverge. This contrasting evolutionary history between real populations and the model suggests that correlations are likely due to extant patterns of connectivity. Empirical populations, on the other hand, may be close to equilibrium because of the relative stability of the ACC, which suggests that populations are relatively old. Additionally, the impact of nonequilibrium conditions may be modest when assessing direct relationships between genetic differentiation and pelagic larval duration (PLD) (Damerau et al. 2014). Nevertheless, it remains important to consider the range of alternative explanations underlying emergent patterns, including possible impacts of recent demographic events.

### Microsatellite analyses

Muscle tissue or fin-clip samples were collected from adult *C. gunnari* and *N. rossii* from South Georgia, South Orkney Islands, Elephant Island, and either Dallman Bay (*C. gunnari*) or South Shetland Islands (*N. rossii*) (Fig.1). Additional samples from *C. gunnari* were obtained from Shag Rocks. A salt-extraction method (Puregene, Gentra Systems) was used to extract the DNA before PCR amplification of microsatellite loci. Eleven microsatellite loci were amplified for *C. gunnari* and 9 for *N. rossii* (detailed in Appendix B) and genotyped on an Applied Biosystems 3130 Genetic Analyser. Genotypes were determined with GeneMapper® (Applied Biosystems, Foster City, CA, USA), using a Genescan 500-ROX size standard. Each allele in every individual was double-checked by two researchers. Loci were screened for null alleles and scoring errors with Micro-checker v.2.2.3 (Van Oosterhout et al. 2004). Sources of error (i.e. excess of homozygote or heterozygote combinations and rare allele combinations) were determined in GenAlex 6.5 (Peakall and Smouse 2012), and offending genotypes were double-checked.

Number of alleles (*N*_a_), observed (*H*O) and expected heterozygosity (*H*S), locus-specific inbreeding coefficients (*F*_IS_), fixation indexes (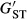) (Nei 1987) and heterozygosity standardized fixation indexes (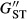) (Meirmans and Hedrick 2011) were all estimated with the package diveRsity (Keenan et al. 2013) for the software environment R. Deviations from Hardy–Weinberg equilibrium (HWE) and linkage equilibrium were tested in GENEPOP V4.0 160 (Rousset 2008) (10 000 dememorizations and 100 batches of 5000 iterations). Global and pairwise 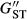 (Meirmans and Hedrick 2011), and 95% confidence intervals based on 1000 bootstraps of individuals among populations, were estimated with diveRsity. To allow comparability with previously published studies while adhering to a single parameter of population differentiation throughout the analysis (*G*_*ST*_), global and pairwise 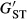, which are comparable in magnitude to estimators of *F*_ST_, were also calculated in GenAlex 6.5. To avoid type II errors, the sequential goodness-of-fit method (Carvajal-Rodriguez et al. 2009) was applied to all *P* values as a correction for multiple testing of all HWE, linkage disequilibrium and differentiation tables.

Empirical genetic structure among sampling locations was visualized through discriminant analysis of principal components (DAPC) (Jombart et al. 2010) in the ADEGENET package (Jombart 2008) for R. As a multivariate methodology that does not make assumptions of HWE or linkage disequilibrium, DAPC first transforms the genotypic data into principal components and then uses discriminant analysis to maximize differentiation among predefined groups (here sampling locations). The number of principal components to be retained was adjusted to include 90% of the cumulative variance, and all discriminant functions were included.

The projected genetic structures (100 iterations) were used to construct synthetic samples. The final allele frequencies of each iteration were resampled to construct one locus for 100 individuals per location per species, creating a synthetic data set of 100 individuals per location/species with 100 loci. The variance among these 100 synthetic loci is thus due to variability in the frequency of certain migration events in each projection, which result in the presence or absence of certain alleles in each population at the end of the simulation. This variance should be similar to that caused by migration and recombination in real genetic markers. The structure of the synthetic data was also visualized through DAPC.

Quantitative comparisons of empirical and projected genetic structure (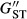) were achieved through regression analyses, and the influence of oceanography on genetic patterns, while allowing for geographic distance, was tested by correlating the empirical and projected residuals of isolation-by-distance regressions.

## Results

### Simulated estimates of connectivity

**Figure 2:**
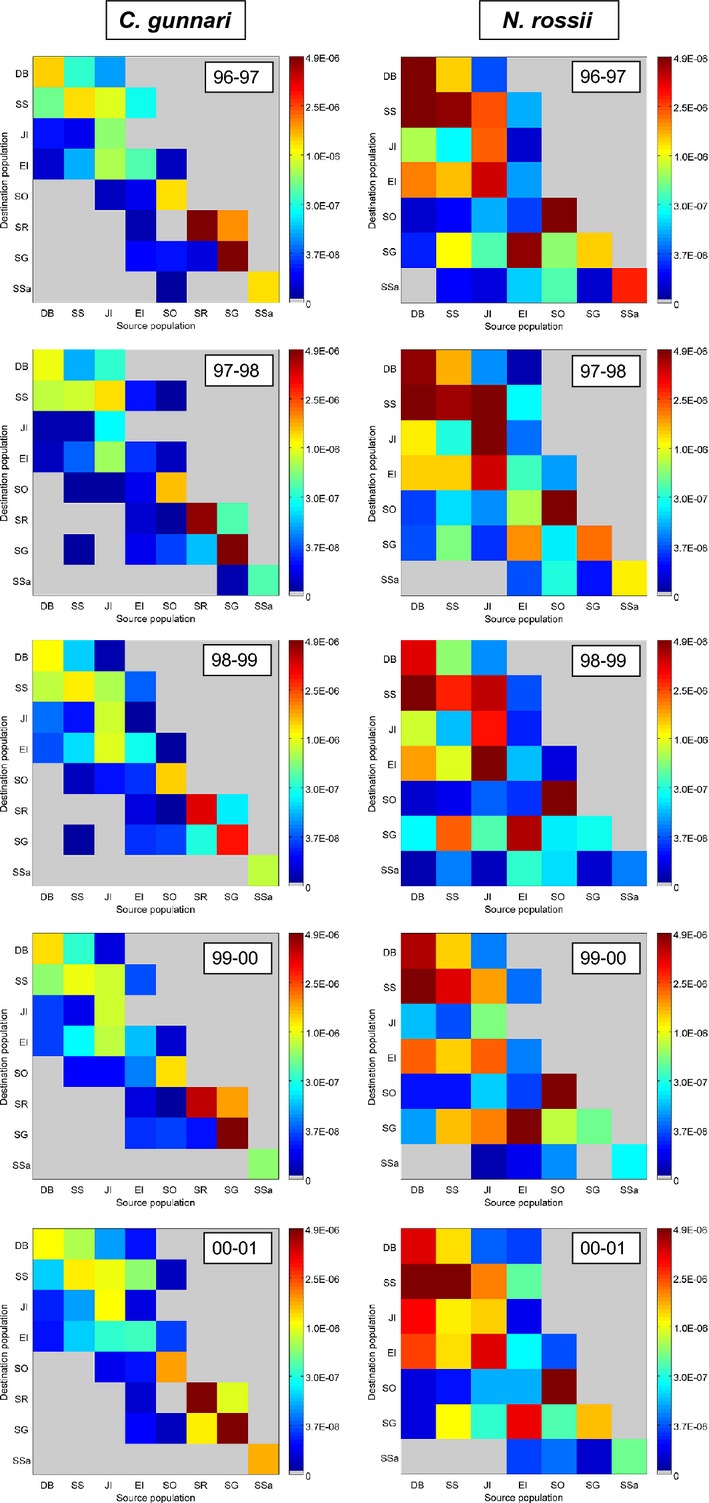
Interannual variability in connectivity matrices *M* for *Champsocephalus gunnari* and *Notothenia rossii*. Population identifiers are Dallman Bay (DB), South Shetland Islands (SS), Joinville Island (JI), Elephant Island (EI), South Orkney Islands (SO), Shag Rocks (SR), South Georgia (SG) and South Sandwich Islands (SSa); Fig.1. Note the colour scale is transformed to allow visualization of the full range of values.

The connectivity matrices indicated wider dispersal of *N. rossii* than that of *C. gunnari*, with higher levels of *N. rossii* transport across the Scotia Sea to islands in the north and east (South Georgia and South Sandwich Islands) relative to *C. gunnari*. Dispersal was highly asymmetric for both *C. gunnari* and *N. rossii* for the five study years, with higher values below the diagonal of the connectivity matrix (Fig.2), indicating unidirectional transport to the north-east across the Scotia Sea in accordance with the dominant north-eastward flows of the ACC. Transport was only bidirectional in the Antarctic Peninsula region, although still generally stronger in the north-eastward direction for both species, and for *C. gunnari* larvae at South Georgia and Shag Rocks. Self-recruitment, indicated by values along the diagonal, was generally stronger at the southern Scotia Sea sites for *N. rossii* than *C. gunnari* due to the lower integrated mortality of *N. rossii* at these locations, but for both species there was a high degree of among-site and interannual variability. Self-recruitment of both species was consistently low at Elephant Island but high at South Orkney Islands. Self-recruitment was also high at South Georgia and Shag Rocks for *C. gunnari* and at Dallman Bay and South Shetland Islands for *N. rossii* (Fig.2).

Although predicted connectivity of both species showed a broad decline with increasing distance between sites, both oceanography and life history modified this relationship. *Champsocephalus gunnari* dispersal was weak across the Scotia Sea, with greater transport predicted between more proximate sites, for example between Shag Rocks and South Georgia, and within the Antarctic Peninsula region. Correlations of the predicted connectivity matrices with rhumb line distance between source and destination sites revealed negative correlations for both species, with correlation coefficients of *r *=* *−0.58 (*P* = 0.001) and *r *=* *−0.46 (*P* = 0.04) for *C. gunnari* and *N. rossii*, respectively. Connectivity was lower than expected from geographic distance for *N. rossii* between proximate sites along the southern Scotia arc, and in particular between the Antarctic Peninsula and South Orkney Islands. *Champsocephalus gunnari* connectivity declined sharply to very low levels for most sites separated by distances of >∼500 km, but there was higher connectivity than expected from geographic distance between South Orkney Islands and South Georgia, and Elephant Island and South Georgia, compared to site pairs of similar separation.

**Figure 3:**
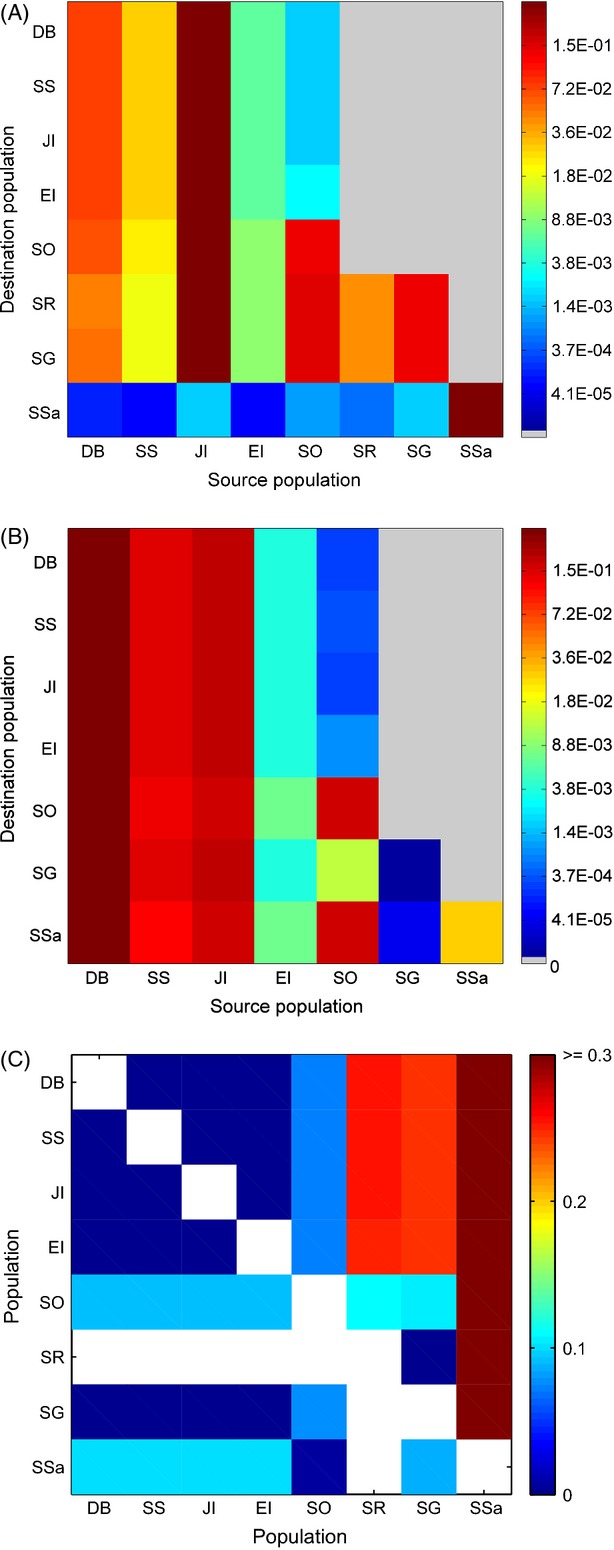
Model projections for *Champsocephalus gunnari* (A, C) and *Notothenia rossii* (B, C) for time integrations chosen by comparison with observed 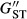: (A, B) probability of finding alleles from each source population (columns) in each destination populations (rows), and (C) genetic differentiation between populations (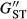); *C. gunnari* above the diagonal, *N. rossii* below the diagonal.

Projections of genetic structure derived from connectivity matrices differed considerably between the two species. The projection model was halted once the maximum projected 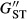 reached the maximum observed value, which occurred after 3519 and 83 time steps for *C. gunnari* and *N. rossii*, respectively. This difference between species corresponds to theoretical expectations of faster approach to equilibrium in populations connected by higher migration rates (Whitlock and McCauley 1999). In *C. gunnari,* allele frequencies in the southern Scotia arc were dominated by immigration from Joinville Island and Dallman Bay (Fig.3A). Joinville Island and Dallman Bay populations also significantly affected allele frequencies at South Georgia and Shag Rocks, even though there was no direct dispersal of larvae predicted between the sites (Fig.2). Rather, gene flow between these sites is a result of stepping-stone transport, via Elephant Island and South Orkney Islands (Fig.2). The South Sandwich Islands were predicted to be relatively isolated, with only low gene flow from other populations in the Scotia Sea, transmitted intermittently via South Orkney Islands and South Georgia (Fig.2). The resultant projected genetic differentiation (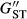) matrix revealed four groupings (Fig.3C, above diagonal): (i) the Antarctic Peninsula region, (ii) South Orkney Islands, (iii) South Georgia and Shag Rocks and (iv) South Sandwich Islands. The level of genetic structuring projected for *N. rossii* was markedly weaker than that of *C. gunnari* (Fig.3C, below diagonal) due to direct dispersal across the Scotia Sea (Fig.2). Allele frequencies across the Antarctic Peninsula region and South Georgia were dominated by alleles originating at Dallman Bay, South Shetland Islands and Joinville Island (Fig.3B), and thus the Antarctic Peninsula sites and South Georgia formed one grouping in the genetic differentiation matrix. However, South Orkney Islands and South Sandwich Islands both had an additional significant contribution of alleles from South Orkney Islands and formed a second grouping.

### Empirical estimates of genetic structure

Empirical patterns of genetic diversity and differentiation inferred from microsatellites differed between *C. gunnari* and *N. rossii*. For *C. gunnari,* the number of alleles per locus ranged from 8 to 39, with observed locus-specific mean heterozygosities from 0.315 to 0.900 and mean observed heterozygosity across loci within samples between 0.690 and 0.710. No locus–sample combination was outside HWE expectations once corrected for multiple testing. One locus pair, *Cgu21* and *Cgu26*, was in significant linkage disequilibrium in Elephant Island after correction, but given that these loci did not show corresponding disequilibria in other samples, we assumed that the loci were not physically linked.

For *N. rossii,* the number of alleles per locus ranged from 10 to 46, with observed locus-specific mean heterozygosities from 0.419 to 0.912 and mean observed heterozygosity across loci within samples between 0.690 and 0.722. One locus, *Nro66*, was significantly out of HWE in two samples, showing heterozygosity deficiency in one sample (SG, *F*_IS_ = 0.218) and heterozygosity excess in the other (SS, *F*_IS_ = −0.007). Locus *Nro66* was thus removed from all further analysis. No locus pairs were in significant linkage disequilibrium once corrected for multiple testing.

Significant population genetic structure among samples was found for *C. gunnari* (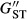 = 0.133; 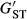 = 0.038; *P* < 0.001), where 12 of 15 sample pairwise comparis-ons were significant after multiple testing correction. The emerging pattern indicated low and nonsignificant values for comparisons within the Antarctic Peninsula, and within South Georgia/Shag Rocks. All comparisons between Antarctic Peninsula, South Orkneys and South Georgia/Shag Rocks were significant (up to 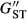 = 0.255; 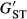 = 0.072; *P* < 0.001). Conversely, no overall significant population genetic structure among samples was found for *N. rossii* (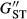 = 0.032; 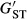 = 0.009; *P* = 0.096). The small size of the South Orkneys sample limited the power of tests for genetic differentiation, and the sample was thus excluded from these tests. The remaining comparisons, including Antarctic Peninsula versus South Georgia, were nonsignificant.

**Figure 4:**
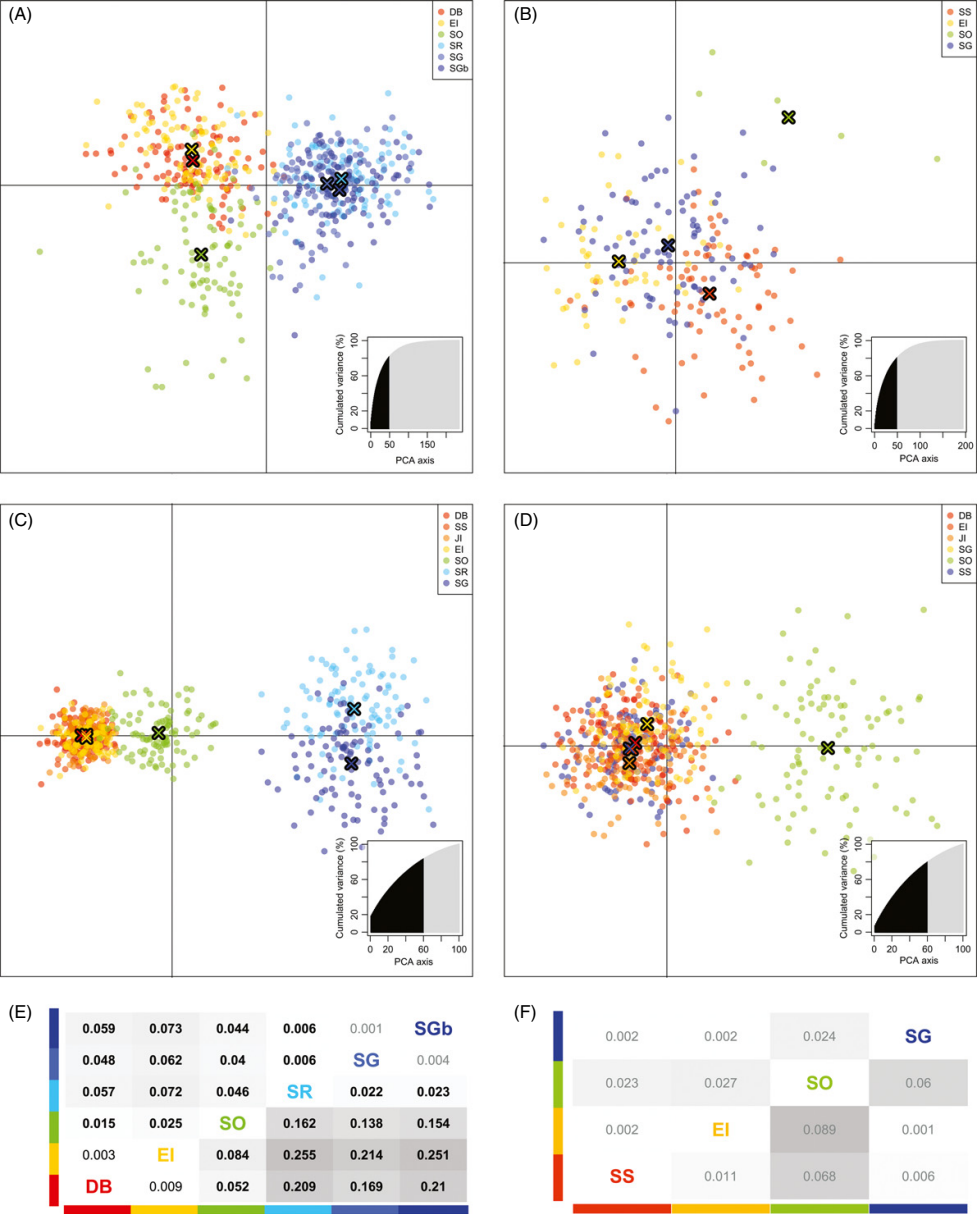
Genetic structure for *Champsocephalus gunnari* and *Notothenia rossii*. Scatter plots of the first two principal components of the DAPC of *C. gunnari* (A, C) and *N. rossii* (B, D) from empirical microsatellite data (A, B) and projected synthetic data (C, D) using sampling locations as prior clusters. Dots represent individuals, and the different colours represent sampling locations: Dallman Bay (DB), South Shetland Islands (SS), Joinville Island (JI), Elephant Island (EI), South Orkney Islands (SO), Shag Rocks (SR) and South Georgia (SG and SGb). The sampling location centroids are represented by coloured crosses. The number of principal components retained and the cumulative variance explained are highlighted in black in the inset. Below the DAPC are tables of pairwise estimates of differentiation (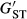 above diagonal and 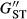 below diagonal) for *C. gunnari* (E) and *N. rossii* (F) from empirical data. Differentiation values are represented as grey heat maps: the darker the grey, the higher the relative value among species comparison. Differentiation values significantly different from zero are in black, and those still significant after correction for multiple testing are in boldface.

The difference in structure patterns between the two species was illustrated through the individual DAPC plots (Fig.4): in agreement with the differentiation tables, the DAPC successfully separated individuals of *C. gunnari* collected in the Antarctic Peninsula (Dallman Bay and Elephant Island) from those collected in South Orkneys and those from Shag Rocks/South Georgia for both the empirical and synthetic data sets (Fig.4A,C). The synthetic data derived from the oceanographic model for *N. rossii* predicted a lack of structure between Antarctic Peninsula and South Georgia (the two most geographically distant sampling locations), but suggested an emergence of genetic differentiation between South Orkneys and the other samples (Fig.4D). The DAPC of the empirical data corroborated the lack of structure between Antarctic Peninsula and South Georgia, and, although the number of South Orkney individuals is small, it positioned them as the most genetically distinct group (Fig.4B).

### Comparison of observed and projected genetic structure

**Figure 5:**
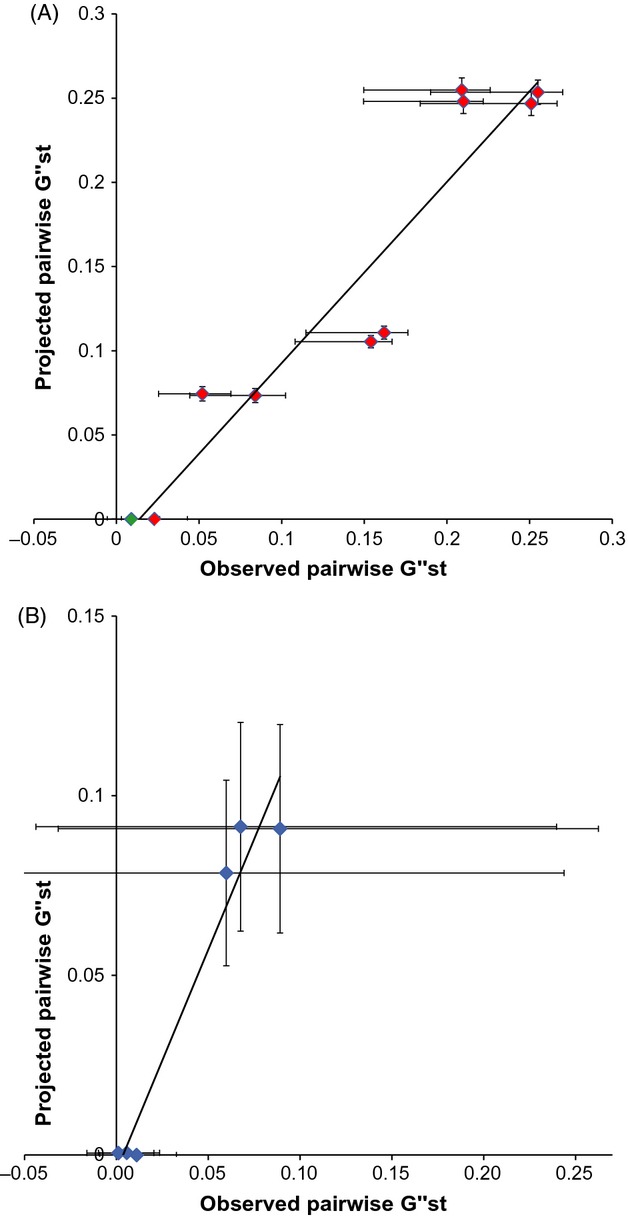
Correlations of empirical and projected pairwise 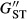 for (A) *Champsocephalus gunnari* and (B) *N. rossii*. Projected 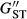 data are the means of 100 repeat simulations using random selection of individual year migration matrices, with standard deviations shown by the vertical error bars. Data points are coloured according to the significance of the empirical 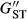: blue values are nonsignificant; green values have *P* < 0.05; and red values are significant after multiple testing correction. Horizontal error bars are the 95% confidence intervals of empirical 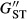 estimates.

For both species, the projected regional genetic structure was in strong agreement with observed structure, with correlation coefficients of *r *=* *0.95 (*P* = 2 × 10^−5^) and *r *=* *0.98 (*P* = 9 × 10^−4^) for *C. gunnari* and *N. rossii*, respectively (Fig.5). Correlations of observed and projected 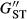 against rhumb line distance between source and destination sites revealed a significant isolation-by-distance pattern in *C. gunnari* in both the observed 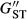 (*r *=* *0.77, *P* = 0.010) and predicted 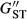 (*r *=* *0.89, *P* = 0.0005). However, neither the projected nor the observed 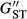 was significantly correlated with distance for *N. rossii* (observed *r *=* *−0.44, *P* = 0.38; projected *r *=* *−0.36, *P* = 0.49). Correlating the residuals from the projected and observed isolation-by-distance relationships allowed consideration of oceanography while allowing for geographic distance. For both species, these correlations were significant, with correlation coefficients of *r *=* *0.93 (*P* = 9 × 10^−5^) and *r *=* *0.98 (*P* = 8 × 10^−4^) for *C. gunnari* and *N. rossii*, respectively. Thus, while both distance between source and destination sites and oceanography significantly affected regional genetic structure in *C. gunnari*, the effect of spatial variability in oceanographic flows was the dominant influence on genetic structure in *N. rossii*.

## Discussion

By combining empirical genetic data and numerical oceanographic, transport and genetic projection models, we have identified important drivers of population genetic structure in two contrasting Antarctic marine fish species. The numerical models projected the observed population genetic structure of both species with a high degree of accuracy, suggesting that parameters used in the model (principally early life history and variability in ocean flows) were a reasonable approximation of reality. Specifically, the difference between the two species in genetic structure and strength in correlations between genetic structure and physical connectivity indicates an important role of early life history on genetic structure, as previously suggested (Cowen and Sponaugle 2009; Galarza et al. 2009; Riginos et al. 2011; Damerau et al. 2014). Such trends in the data were apparent despite ongoing controversy in the absolute measures of key life-history events in these species, such as timing and location of egg development, hatching times and dispersal period (Damerau et al. 2014). Such traits are inherently difficult to quantify due to geographic isolation and the extreme temporal and spatial patchiness in egg and larval distribution of Antarctic marine species (North and Murray 1992). Regional differences in spawning times and rates of development (Everson 1984; North 2005) can add additional variance on local dispersal and projected recruitment rates. Despite such uncertainties, however, our results represent a rare demonstration of the effect of species biology and environmental factors in determining population genetic structure.

### Patterns and drivers of genetic structure

Broadly speaking, populations of *N. rossii* are genetically more similar to each other than those of *C. gunnari*. With a total planktonic phase of approximately 7 months, more than twice that of *C. gunnari*, larvae spawned at the Antarctic Peninsula, have the potential to recruit as far as to the South Georgia population. However, the population at South Orkney Islands appears more genetically differentiated, in contradiction to a simple isolation-by-distance trend. This observation was derived from a small sample at South Orkney Islands (*n *=* *6) which may inflate levels of genetic differentiation. Nevertheless, the sample was retained because the pattern corresponded to the modelled differentiation of the population at South Orkney Islands. Such differentiation is potentially due to the dominant oceanographic flows in the region, which circumvent the South Orkney Islands and directly connect the Antarctic Peninsula with South Georgia. The relative isolation of South Orkney Islands is corroborated by drifter data, which reveal a northward flow of Weddell Sea water across the South Scotia Ridge between Elephant Island and the South Orkney Islands, acting as a dynamic barrier to exchange between populations at the Antarctic Peninsula and South Orkney Islands (Thompson et al. 2009; Thompson and Youngs 2013).

By contrast, with a shorter planktonic phase, *C. gunnari* exhibits greater genetic structuring across the Scotia Sea, in broad agreement with the expected pattern from a consideration of isolation by distance. Because of its shorter planktonic phase, direct dispersal between the Antarctic Peninsula and South Georgia is less likely. Instead, the numerical modelling identified stepping-stone transport pathways from the Antarctic Peninsula to Elephant Island and South Orkney Islands, and finally to South Georgia. While the oceanographic barrier limiting larval exchange between the Antarctic Peninsula and South Orkney Islands is again apparent in the model projections, connectivity between the Antarctic Peninsula and South Georgia is even weaker due to the relatively short planktonic larval phase. Thus, the stepping-stone pathways arise as a result of the directionality and speed of the dominant oceanographic flows in the region, and the distance these flows can transport larvae during their 3-month planktonic phase. Such dispersal mechanisms are especially critical in environments such as the Antarctic where suitable habitats to complete development of life-history stages are highly fragmented.

Although the population structure of both species was strongly correlated with oceanographic patterns, only *C. gunnari* showed a classical isolation-by-distance pattern, suggesting that in species with shorter pelagic periods, geographic distance may be a major barrier to gene flow. Such assertions correspond well to reports of strong isolation-by-distance patterns in other marine species with PLD of a few months or less (Buonaccorsi et al. 2002, 2005; Cunningham et al. 2009). However, in many other species, including *N. rossii*, simple geographic distance may be an unsuitable predictor of genetic structure, possibly explaining the relatively weak relationship between PLD and isolation-by-distance slope (Selkoe and Toonen 2011). Not surprisingly, main drivers of connectivity differ among species, even when larvae are dispersed passively. Such species differences are relevant not only for evolutionary research, but also for the design of marine-protected area networks (Curley et al. 2013) and prediction of species responses to global climate change (Lett et al. 2010).

Similar patterns of genetic differentiation were found in a recent comparison of *C. gunnari* and *Chaenocephalus aceratus* in the same geographic region (Damerau et al. 2014). Marked genetic structuring was observed in both species, although *C. gunnari* exhibited higher genetic differentiation compared to *C. aceratus*. As similar pelagic periods of about 1 year were assumed in both species, Damerau et al. (2014) concluded that predicted dispersal capacity based on life-history traits did not explain patterns of genetic structuring. Instead, they attributed differences in genetic differentiation between the species to a mix of factors such as differences in generation times, nonequilibrium conditions and associated demographic effects, as well as potential constraints to gene flow due to local adaptation. In contrast, our study showed that oceanographic models predicted genetic structure accurately, implying (i) that the influence of demographic history of these species on their genetic patterns is limited, (ii) that local adaptation restricts reproductive success on immigrants, and thus gene flow, equally in all locations, if at all, and (iii) that the planktonic larval period of 3 months may be more accurate in predicting dispersal than the 1-year pelagic phase postulated in Damerau et al. (2014).

Demographic histories of studied populations are likely to affect patterns of genetic differentiation, although the relative importance of historical and extant forces is often difficult to assess (Marko and Hart 2011). Although the ACC was established at least 6 million years ago (Barker et al. 2007), the Pleistocene glacial maxima had significant effects on Antarctic fishes and their shelf habitat. Sea level reductions and extended ice coverage likely eliminated most shelf habitats during the past glacial maximum about 20 000 years ago (Anderson et al. 2002). Benthic shelf habitats in the Weddell Sea likely became ice-free only about 7000 years ago, and so the populations surveyed here may be younger than originally assumed. Indeed, many Antarctic fish species show genetic signals of population expansion likely associated with that ice retreat (Patarnello et al. 2011), with stronger climatic effects on benthic than on pelagic fish species (Janko et al. 2007). Nevertheless, populations of *C. gunnari* and *N. rossii* may be sufficiently close to migration–drift equilibrium to allow meaningful inferences in gene flow from genetic data. First, the signal for population expansion in *C. gunnari* is relatively weak, possibly also due to the limited power of the marker (Damerau et al. 2014). Second, both species follow an isolation-by-distance model, where distance is both geographic and oceanographic in *C. gunnari* and only oceanographic in *N. rossii*. Both empirical (Crispo and Hendry 2005) and theoretical (Slatkin 1993) evidence suggest that isolation-by-distance patterns are achieved much faster than equilibrium in a standard island model of migration. Finally, the close correspondence between genetic patterns predicted from the oceanographic model and observed in wild populations suggests dispersal of the planktonic egg and larval stages is the main driver of genetic differentiation.

The strong correlation between projected and realized gene flow also implies that immigrants either had equal reproductive success to resident fish or that the reduction in immigrants’ fitness due to local adaptation was the same everywhere and independent of the source of immigrants. Increasing evidence that marine fishes display a higher than expected localized scale of local adaptation (Hauser and Carvalho 2008; Limborg et al. 2012; Hemmer-Hansen et al. 2013) indicates that successful dispersal of larvae may be constrained in some circumstances by selection. Documented evidence of high selective mortality (Planes and Lenfant 2002; Veliz et al. 2006; Vigliola et al. 2007) and low reproductive success (Peterson et al. 2014) lends support to the role that local adaptation can play in structuring marine fish populations. If so, genetic differentiation may be determined more by isolation by adaptation (local adaptation) than isolation by distance (dispersal), with populations in the most heterogeneous rather than least connected habitats showing the highest genetic differentiation (Nosil et al. 2009). Although not explicitly tested, there was little evidence for such isolation by adaptation, despite large differences in environmental conditions between the Antarctic Peninsula and South Georgia (Murphy et al. 2013). However, the strong flow of the ACC implies unidirectional dispersal across the Scotia Sea, which characterizes the Antarctic Peninsula as a source population with little immigration once depleted and South Georgia as a sink population. If so, the South Georgia population may be suboptimally adapted to its local conditions because of high ‘migration load’ from immigrants (Lenormand 2002), which may reduce selection against immigrants and may limit adaptation to further environmental change. Such hypotheses could be tested further by employing both putatively neutral and selected markers (Limborg et al. 2012; Nielsen et al. 2012).

Previous studies of genetic differentiation in Scotia Sea populations of *C. gunnari* have assumed long dispersal periods of about 1 year (Damerau et al. 2012, 2014) and have included dispersal of eggs, planktonic larvae and the later pelagic stages. However, there is some debate regarding the likelihood of dispersal during each of these stages. Recent molecular studies (E. M. Fitzcharles, BAS, unpublished data) have confirmed previous observations (Everson et al. 2001) that *C. gunnari* lay adhesive demersal eggs that form clusters, and dispersal during the egg phase is therefore highly unlikely. Although there is some uncertainty regarding the length of the planktonic larval phase, which is likely to vary with local environmental conditions such as temperature, data from South Georgia and Kerguelen Island suggest a planktonic larval phase of approximately 3 months (Duhamel 1995; Belchier and Lawson 2013), and larval growth rates at the South Shetland Islands are within the range observed at South Georgia and Kerguelen (La Mesa et al. 2013). After such time, juveniles, while still pelagic, are found at highest densities in shallow inner shelf areas where abundance of their preferred prey is greatest (Atkinson et al. 1990). During the pelagic juvenile phase, animals undergo horizontal and vertical migration (Frolkina 2002) and are therefore able to maintain position within the nursery areas on the shelf. Consequently, dispersal of juveniles by ocean currents is likely to be negligible. Here, we considered dispersal to occur primarily during the planktonic larval phase and achieved good agreement between projected and empirical genetic differentiation. Such congruence suggests strongly that dispersal of *C. gunnari* does indeed occur primarily during the relatively short planktonic larval phase of approximately 3 months.

### Broader implications

An integrative and quantitative approach involving oceanography, life history and population genetics moves significantly beyond qualitative measures typically employed to assess the impact of life history on genetic structuring, such as PLD, which has often displayed incongruent associations (Cowen and Sponaugle 2009; Selkoe and Toonen 2011). The frequent lack of fit with expectations endorses the complexity and ambiguity of factors impacting recruitment, as well as the oversimplification of proxies such as *F*_ST_ that may fail to account for the impact of variance in key parameters such as effective population size on correlations between PLD and *F*_ST_ (Damerau et al. 2014). A variety of additional factors may contribute to such deviations from predictions, including oceanographic variability in dispersal pathways, timing and location of hatching and larval release, larval behaviour and mortality, and local adaptation (Faurby and Barber 2012). However, the strong correspondence between oceanographically informed individual-based simulations and microsatellite-derived estimates of genetic differentiation detected here indicates a confluence of demographic and evolutionary timescales. Such correspondence emphasizes the high ecological relevance of short-term estimates of connectivity in our system, thereby yielding a potential baseline for predicting impacts of ecological disturbance. However, differences in patterns of connectivity across the 5 years suggest that deterministic predictions based on a single year may have been greatly misleading (Fig.2) and thus confirm previous assertions that temporal stochasticity in ocean current patterns has marked impacts on the ecological and genetic dynamics of metapopulations (Watson et al. 2012).

Our study also demonstrates that even in species with long pelagic life-history stages, populations may be connected by only limited dispersal, leading to reduced demographic synchrony and genetic differentiation. Such independent populations may be significant for management, both in the short (fisheries management) and in the long term (adaptation to global climate change). Independent, locally adapted populations may react asynchronously to environmental perturbation (Hilborn et al. 2003) and may thus increase resilience of the entire metapopulation to natural and anthropogenic disturbance (portfolio effect, Schindler et al. 2010). On the other hand, asymmetric dispersal within such a metapopulation may result in a source–sink population dynamic with maladapted sink populations that do not contribute to the overall productivity of the metapopulation (Lenormand 2002). Such patterns of dispersal are particularly relevant for the design of MPA networks (Crowder et al. 2000). In our system, for example, an MPA at South Georgia would have little benefit for populations at the Antarctic Peninsula or at the South Orkney Islands.

The projections of genetic structure were highly sensitive to the underlying oceanographic flows, which has potentially important implications for genetic connectivity in a changing world. There is currently significant uncertainty concerning the response of circulation patterns in the Scotia Sea, and in the Southern Ocean more generally, to climatic changes. Warming of the Southern Ocean on decadal timescales has been demonstrated clearly (Boning et al. 2008; Gille 2008), and it has been theorized that a significant part of this warming signal is due to a poleward migration of the frontal features of the ACC concurrent with a contraction of the atmospheric polar vortex (Sokolov and Rintoul 2007; Meijers et al. 2011). However, diagnosing the changing positions of fronts can be problematic, even using remotely sensed data from satellites (Graham et al. 2012; Gille 2014), and other factors are also believed to contribute to the warming observed (Hogg et al. 2008). One of the key modes of climate variability in the region, the Southern Annular Mode (SAM), is exhibiting an increasingly positive trend, which is at least partly attributed to anthropogenic causes (Thompson and Solomon 2002; Marshall et al. 2004). Positive SAM is associated with increased westerly winds over the circumpolar Southern Ocean (Marshall et al. 2006), with an associated reduction in westward oceanic transport in the Antarctic Peninsula region and an increase in transport from the Antarctic Peninsula to South Georgia (Renner et al. 2012). Such transport patterns could reduce bidirectional mixing of populations in the Antarctic Peninsula region and increase population separation and consequent genetic structuring between populations on either side of the peninsula (e.g. Joinville Island and Dallman Bay). Conversely, increased transport across the Scotia Sea could enhance gene flow between the Antarctic Peninsula and South Georgia, thus reducing genetic differentiation between these populations and potential for local adaptation. Such influences could therefore have a significant impact on the genetic structure and resilience of fish populations in the Scotia Sea. However, further progress is required to better understand the past and future changes in circulation that are caused by variable climatic forcings, and the subsequent impacts on population genetic structure.

## Summary

Here, we have shown that key biological characteristics of the early life-history stages of two Antarctic fishes, when integrated into individual-based models of connectivity and genetic projection models, can yield informative and accurate projections of population genetic differentiation. *Champsocephalus gunnari*, with a planktonic phase that is approximately only half the duration of *N. rossii*, exhibited higher levels of genetic structuring, with model results suggesting both isolation-by-distance and stepping-stone models of gene flow, dependent upon the geographic separation of populations and directionality of dominant oceanographic flows. The extent of interannual variability in oceanographic–population genetic structure projections across 5 years endorses the need to incorporate, where possible, a temporal series of samples for comparison, on both localized and regional scales. Population connectivity effected by planktonic dispersal is evidently a key determinant of population recruitment and persistence of Antarctic fishes that inhabit highly seasonal and fragmented environments. Such findings emphasize the vulnerability of such populations to factors likely to disrupt population connectivity, including the impact of elevated temperatures on egg and larval development and planktonic duration, habitat change and over-exploitation. There remains an urgent need to better define the physiological ecology of larvae across changing environments, together with directed functional genomic analyses of the patterns and scale of local adaptation.

## Figures and Tables

**Figure 6:**
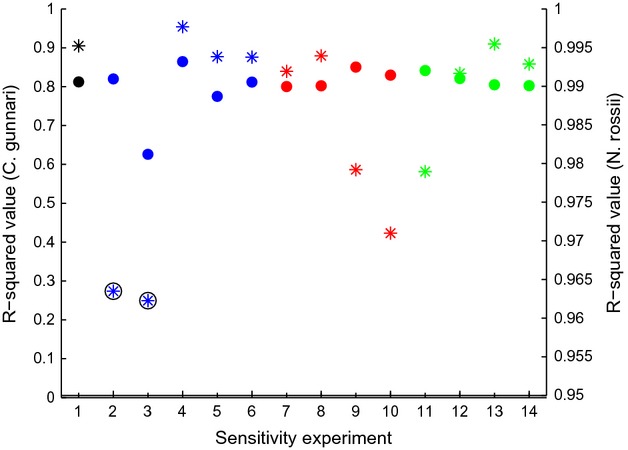
R-squared values from a comparison of projected and empirical 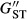 values for the sensitivity experiments detailed in Table1. Results for *Champsocephalus gunnari* (stars) are plotted on the left hand *y*-axis, while those for *Notothenia rossii* (filled circles) are plotted on the right hand *y*-axis. Nonsignificant correlations (*P* > 0.05) are circled. The symbols are coloured according to the parameter under test: connectivity matrix (blue), mortality rate (red) and birth rate (green). For comparison, the black symbols indicate the *r*-squared values obtained with the ‘best guess’ choice of mortality and birth rates, and with the mean connectivity matrix.

**Figure 7:**
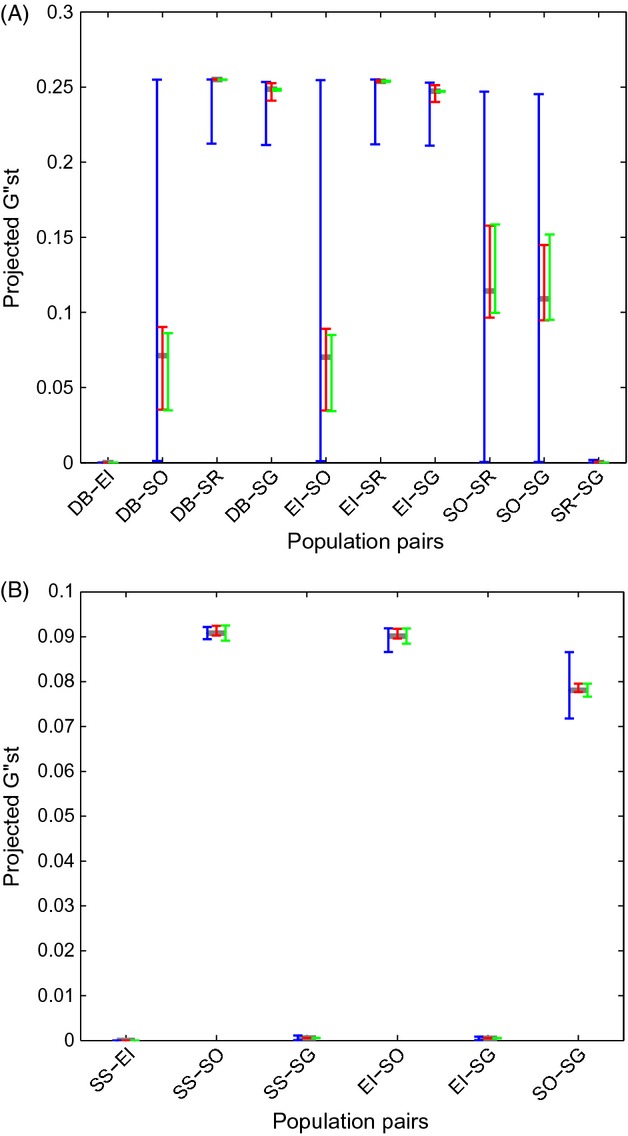
Sensitivity of projections of pairwise 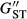 to model parameterization (Table1) for (A) *Champsocephalus gunnari* and (B) *Notothenia rossii*. Grey lines are the ‘best guess’ projection. Error bars show the range of values obtained from adjusting the connectivity matrix (blue), mortality rate (red) and birth rate (green).

## Data archiving statement

Sequencing information has been deposited in the European Molecular Biology Laboratory (EMBL), accession numbers FM878464–FM878589 and LN614714–LN614727 (Appendix B). Full genotypic data are stored on the Molecular Ecology and Fisheries Genetics Laboratory (MEFGL) database at Bangor University. Output from the modelling work is archived at the British Antarctic Survey.

## Appendix A

### Genetics projection model sensitivity analysis

#### Methodology

The sensitivity of the model projections to the choice of birth rate, mortality rate and variability in the underlying oceanography was assessed. An initial simulation, using the ‘best guess’ birth and mortality rates detailed in the Methodology, and the mean connectivity matrix calculated by averaging the 5 year-specific matrices, was performed for comparison purposes. Birth rates were varied between minimum and maximum values detailed in Kock and Kellermann (1991), thus between 500 and 12 400 per individual for *Champsocephalus gunnari* and between 7600 and 52 000 per individual for *Notothenia rossii*. A range of mortality rates was estimated from eqn (1) by applying a temperature range of −1 to +3°C. To assess the sensitivity of model projections to variability in the underlying oceanography, simulations were performed with each of the 5 year-specific matrices. The parameterization of each sensitivity experiment is summarized in Table1. Two sets of simulations were performed to assess model sensitivity, with model parameters changed sequentially in each case: (i) the model was halted after the same number of time steps as the ‘best guess’ simulation, and (ii) the model was halted once the projected maximum  value matched that observed. For each simulation, the change in the projected  values was assessed, and the effect of this change on the correlation with observed genetic structure was evaluated.

**Table A1:** Parameterization of sensitivity experiments

Experiment number	Parameterization
1	‘Best guess’
2	1996 connectivity matrix
3	1997 connectivity matrix
4	1998 connectivity matrix
5	1999 connectivity matrix
6	2000 connectivity matrix
7	Low mortality (*T* = −1°C)
8	Mid-low mortality (*T* = 0°C)
9	Mid-high mortality (*T* = 2°C)
10	High mortality (*T* = 3°C)
11	Low birth
12	Mid-low birth
13	Mid-high birth
14	High birth

#### Results

Considering first the simulations with an imposed number of time steps (Case 1), projected  values for *C. gunnari* showed a high degree of sensitivity to variability in the chosen birth and mortality rates, and to the connectivity matrix, for the majority of site pairs. The two exceptions to this were South Georgia/Shag Rocks and Dallman Bay/Elephant Island, both of which had extremely low  values in all simulations. However, although the absolute  values were sensitive to the model parameterization, the relative population genetic structure compared well with the observed structure for the majority of simulations. High values of *r*^2^ (Fig.6) and significant correlations (*P* < 0.05) were obtained for the full range of birth and mortality rates, with weaker, but still significant, correlations associated with lower birth rates and higher mortality rates. However, the ability of the projection model to predict the observed genetic structure was impacted by the choice of connectivity matrix, with poor correlations obtained for year-specific matrices from 1996 and 1997, indicating a high degree of sensitivity to variability in the oceanographic flows. This is further demonstrated by the second set of sensitivity analyses, where the number of model time steps was chosen by matching the maximum projected  value with that observed. Simulations using connectivity matrices from 1996 and 1997 again failed to project the observed genetic structure, with a wide range of  values projected for some site pairs (Fig.7A). However, high values of *r*^2^ and significant correlations were obtained for all other sensitivity experiments, suggesting that the projection model for *C. gunnari* is sensitive to the underlying oceanographic flows, but relatively insensitive to the choice of birth and mortality rates.

Projected  values (Case 1) for *N. rossii* showed a mixed response with half the site pairs not sensitive to the underlying model parameterization: South Shetland Islands/Elephant Island, South Shetland Islands/South Georgia and Elephant Island/South Georgia. These site pairs had very low values of  in all simulations. The remaining site pairs were sensitive to variability in the mortality rate, and the choice of connectivity matrix, but were less sensitive to birth rate. However, the  values projected in the second set of experiments showed very little variability for all site pairs (Fig.7B). In addition, the projected population genetic structure was highly correlated with the observed structure for all the sensitivity experiments (Fig.6); thus, the projection model for *N. rossii* can be considered insensitive to the underlying model parameterization.

#### Summary

The projection model for *C. gunnari* is more sensitive to the model parameterization than that for *N. rossii*. However, even though absolute values of  in both species were sensitive to input parameters, the projected population genetic structures were significantly correlated with those observed for the majority of simulations, demonstrating the overall robustness of the genetics projection model. The biggest influence on projected  was the connectivity matrix, thus highlighting the importance of adequate representation of the underlying oceanographic flows for genetic projections, and the potential for future impacts on population genetic structure due to changes in circulation caused by large-scale variability in climatic forcing.

## Appendix B

### Microsatellite sequencing and locus characteristics for *Champsocephalus gunnari* and *Notothenia rossii*

All source primers were developed according to the following protocol. An enriched microsatellite library was prepared separately for *C. gunnari* and *N. rossii*. The individuals used to create the libraries were for *C. gunnari* (*n* = 11) and for *N. rossii* (*n* = 9) both species sampled at South Georgia in 2002. The two genomic libraries were constructed using the method of Armour et al. (1994), and both were enriched for the following di- and tetranucleotide microsatellite motifs: (GT)_n_, (CT)_n_, (GTAA)_n_, (CTAA)_n_, (TAAA)_n_, (TTTC)_n_ and (GATA)_n_ and their complements, which had been denatured and bound to HybondN+ nylon membrane. Transformant colonies were screened for the presence of a repeat region. A total of 68 *C. gunnari* and 72* N. rossii* microsatellite sequences were isolated and submitted to the EMBL sequence database (*C. gunnari*: FM878464–FM878524 and LN614714–LN614720; *N. rossii*: FM878525–FM878589 and LN614721–LN614727). Primer sets were designed using PRIMER3 v0.4.0 (Rozen and Skaletsky 2000). A selection of these newly isolated loci was used in this study (Table2). Locus-specific characteristics are reported in Table3, and sample-specific locus characteristics are detailed in Table4.

**Table B1:** Repeat motif, primer sequences and PCR annealing temperature for *Champsocephalus gunnari* (*C. g*.) and *Notothenia rossii* (*N. r*.) microsatellite loci

	Clone	Source	Repeat motif	R primer	L primer	Annealing °C
*C. g. locus*						
Cgu06	Cgu25C04b	FM878469	(AC)_9_(AT)_3_(AC)_2_G(TA)_2_	GATCGGCCGTAATCAAAGGAGAG	TCAGTCCAAGCCAAGATTCAAGG	63
Cgu12	Cgu25D12	FM878475	(TG)_23_	CCAGGCTTACCTCACCAAGCAC	TGTACCACCTGGCCATTCTGTG	63
Cgu16	Cgu25F03	FM878479	(AC)_8_GCACGG(CA)_3_TG(AA)_2_	CCCTCTCTTTACCCATTCCTC	CCATCATTTATGGCTGATTCC	58
Cgu18	Cgu25H04	FM878481	(AC)_4_(AT)_2_(AC)_5_TTCT(AC)_11_	TCTGCGGACTCCTCTGACAGC	TCCTCCGGAGCGAGCATAAG	60
Cgu21	Cgu26A04	FM878484	(CA)_7_CGCACG(CA)_6_	CTCTGCTGCGTCCGTCTGTC	AGCCGCCTCTCTACACACAGC	63
Cgu26	Cgu26D11	FM878489	(TG)_11_	TCGAGTCTCAGTTCGGCATCC	CCAGTGCTCAAAGACGCTTTCC	63
Cgu30	Cgu26E05	FM878493	(TG)_13_CGTGCGTGCATGTGC(GT)_2_GC(GT)_5_	AGACGAGGCTCCTCGCACAC	TGGGTCGCCTCCTCTCTCAC	60
Cgu32	Cgu26G04	FM878495	(GT)_15_	AGACAGCTCTGACACGCTCAGG	TATACACCCAGTCAGAGACAAAGTCACAG	60
Cgu34	Cgu26G12b	FM878497	(GT)_17_	CGTTGCTCAGCCAGTTTCTCC	GGGCCAGTGAGGATTGTTGG	63
Cgu44	Cgu27E07	FM878507	(TG)_14_	TTCCTGCACTCCTGCTGCTG	CTTAAGAGCATTGACTTTCTGTGTGAGC	63
Cgu50	Cgu27H09	FM878513	(AC)_10_	TGGCGACACAGCTTGGTATAG	GATCCCAGTCCATGTTGCAG	63
Cgu70	Cg4A5	*	(CA)_25_	GGTTTGCCCTCTCCCATGA	GGTTGTTACAGTGTGCCGTTTG	61
*N. r. locus*						
Nro03	Nro15D05	FM878527	(CA)_3_CT(CA)_2_TGCACG(CA)_6_	TCCCAAGCTCTGGCAATGTG	CCAAACAACACGGGTAAGTCAGC	63
Nro11	Nro16A11	FM878535	(GT)_10_	GCTGGGTGGAGAGGTGGAGA	TGCATGGGTAATGACCCTGTTG	63
Nro27	Nro17E09	FM878551	(CT)_2_(CA)_11_	TTAATATGCCGCCAGCAAAGG	TCCGACAACTTCAGCGCATATC	63
Nro41	Nro18D02	FM878537	(CA)_10_	AAGAGCGCACCTTCCACACC	GTGCAATGCAATGTGTGACCAG	60
Nro46	Nro18G03	FM878570	(C)_14_(AC)_11_	GGAATCGATTAGCTCGGGTTTATTTG	CAACCATTAGCCAATAACCAACCAAG	63
Nro50	Nro20B11	FM878574	(GATA)_3_TAAATA(GATA)_2_TA(GATA)_3_GA(CAGA)_12_	TCATACATGTTCCAATTGCTG	TTGAACTACTACATATGGATGGATG	56
Nro66	Nro81C01	LN614721	(TC)_6_CT(TC)_4_CT(TC)_5_TT(TC)_7_	CGGCTGTGCACTGACACTGAG	GGGCTGATAGGGCAGAGCAAC	58
Nro68	Nro81F04	LN614723	(GT)_9_	GTCTGGCAGGTGTCGAGCAG	CTGGAGGATATCCACTGACACAGC	60
Nro69	Nro81H06	LN614724	(GT)_5_GCT(TG)_4_CT(TG)_4_CT(TG)_4_CGTGC(GT)_8_	TTGAGCATAGAGGTGCTGCAGTG	TGACCGCGTCTCTCCATCAG	60
Cgu69	Cg1A5	*	(TG)_42_	TGGATGTTGGTGATGAGGAC	TCAGGATGTGATAGAGAGCACTTT	60
Cgu70	Cg4A5	*	(CA)25	GGTTTGCCCTCTCCCATGA	GGTTGTTACAGTGTGCCGTTTG	60

* H. R. Wilcock, unpublished data.

**Table B2:** Locus-specific characteristics: number of individuals genotyped (*N*), number of alleles detected (*A*), the allele range in base pair (range), observed (*H*_o_) and expected (*H*_e_) heterozygosities, global fixation indices ( and ) and inbreeding coefficient (*F*_IS_)

	*N*	*A*	Range	*H*_o_	*H*_e_			*F*_IS_
*C. g. locus*								
Cgu06	568	15	96–150	0.730	0.769	0.018	0.075	0.050
Cgu12	556	28	229–291	0.868	0.890	0.019	0.178	0.025
Cgu16	511	11	234–262	0.718	0.715	0.007	0.024	−0.003
Cgu18	544	9	231–255	0.413	0.434	0.158	0.261	0.047
Cgu21	563	8	221–247	0.315	0.339	0.030	0.040	0.070
Cgu26	552	39	231–350	0.717	0.762	0.052	0.215	0.060
Cgu30	546	29	216–286	0.691	0.714	0.041	0.141	0.032
Cgu32	557	8	135–185	0.614	0.620	0.025	0.064	0.009
Cgu34	552	21	182–224	0.764	0.799	0.028	0.141	0.045
Cgu44	551	24	171–217	0.792	0.822	0.064	0.359	0.036
Cgu50	556	20	223–279	0.729	0.780	0.040	0.181	0.065
Cgu70	572	35	135–219	0.900	0.900	0.006	0.063	0.001
*N. r. locus*								
Nro03	202	11	247–266	0.420	0.426	0.005	0.009	0.004
Nro11	213	14	175–203	0.675	0.630	0.011	0.031	−0.076
Nro27	191	15	213–259	0.426	0.385	0.017	0.026	−0.102
Nro41	216	22	140–190	0.911	0.865	0.001	0.008	−0.044
Nro46	193	15	225–259	0.628	0.690	0.037	0.151	0.129
Nro50	184	24	136–240	0.912	0.915	0.000	0.004	0.016
Nro66	200	10	179–219	0.620	0.619	0.001	0.003	−0.093
Nro68	195	24	228–286	0.886	0.887	0.011	0.129	−0.018
Nro69	207	23	248–316	0.660	0.652	0.014	0.047	−0.028
Cgu69	189	46	220–314	0.903	0.955	0.003	0.097	0.062
Cgu70	188	14	130–180	0.672	0.712	0.002	0.006	0.038

**Table B3:** Sample-specific locus characteristics. Species and location of each sample are indicated in the top left of each subtable; locus names head each column; *N* = number of individuals successfully genotyped; *A* = number of alleles detected; *H*_o_ = observed heterozygosity; *H*_e_ = expected heterozygosity; *F*_IS_ = inbreeding coefficient; HWE(*P*) = associated probability value of conformation with Hardy–Weinberg equilibrium expectations (HWE). Bold *P* values indicate significant deviations from HWE after multiple testing correction

	Cgu06	Cgu12	Cgu16	Cgu18	Cgu21	Cgu26	Cgu30	Cgu32	Cgu34	Cgu44	Cgu50	Cgu70
*C. gunnari* (DB)												
*N*	102	104	102	98	104	103	103	102	91	98	102	99
*A*	7	21	9	5	5	24	17	7	11	17	12	19
*H*_o_	0.78	0.89	0.66	0.31	0.34	0.77	0.64	0.54	0.71	0.74	0.8	0.87
*H*_e_	0.77	0.92	0.71	0.34	0.37	0.81	0.76	0.54	0.76	0.82	0.84	0.9
*F*_IS_	−0.0185	0.0229	0.0751	0.1012	0.092	0.0576	0.1541	−0.0012	0.0658	0.0952	0.0485	0.0319
HWE(*P*)	0.822	0.196	0.041	0.229	0.269	0.266	0.025	0.099	0.241	0.042	0.485	0.918
*C. gunnari* (EI)												
*N*	93	84	82	86	86	84	86	89	90	87	86	92
*A*	6	19	9	5	5	28	16	7	10	16	9	19
*H*_o_	0.71	0.82	0.68	0.22	0.42	0.77	0.76	0.64	0.7	0.78	0.83	0.89
*H*_e_	0.71	0.9	0.68	0.21	0.44	0.84	0.75	0.55	0.76	0.78	0.83	0.9
*F*_IS_	−0.0057	0.0831	−0.0075	−0.0431	0.0449	0.0799	−0.0086	−0.1613	0.0818	−0.002	0.0089	0.008
HWE(*P*)	0.511	0.089	0.058	0.519	0.257	0.458	0.859	0.284	0.431	0.391	0.917	0.747
*C*. *gunnari* (SO)												
*N*	87	84	77	86	82	89	73	82	91	87	81	91
*A*	8	19	10	6	4	18	12	7	17	17	10	19
*H*_o_	0.75	0.83	0.74	0.36	0.22	0.74	0.78	0.68	0.78	0.75	0.77	0.9
*H*_e_	0.78	0.88	0.75	0.39	0.23	0.71	0.71	0.64	0.77	0.83	0.8	0.86
*F*_IS_	0.0431	0.0528	0.0107	0.0864	0.0444	−0.0472	−0.0931	−0.0664	−0.0087	0.0989	0.0484	−0.0467
HWE(*P*)	0.368	0.033	0.114	0.170	0.377	0.854	0.782	0.387	0.046	0.332	0.638	0.806
*C*. *gunnari* (SR)												
*N*	94	99	76	96	100	98	97	99	96	100	101	102
*A*	10	16	7	7	6	16	17	7	18	15	13	26
*H*_o_	0.66	0.92	0.79	0.56	0.35	0.7	0.58	0.65	0.83	0.86	0.62	0.94
*H*_e_	0.77	0.9	0.75	0.55	0.37	0.76	0.6	0.7	0.86	0.84	0.77	0.92
*F*_IS_	0.1464	−0.027	−0.0559	−0.0197	0.0649	0.0695	0.0381	0.0728	0.0266	−0.0185	0.1899	−0.0213
HWE(*P*)	0.152	0.584	0.780	0.948	0.469	0.084	0.321	0.610	0.061	0.793	0.032	0.817
*C. gunnari* (SG)												
*N*	96	92	86	84	95	92	93	91	89	92	94	92
*A*	8	20	9	5	6	17	20	5	18	16	17	19
*H*_o_	0.69	0.85	0.72	0.48	0.29	0.68	0.7	0.55	0.76	0.84	0.7	0.87
*H*_e_	0.78	0.88	0.69	0.55	0.32	0.74	0.75	0.58	0.81	0.83	0.76	0.92
*F*_IS_	0.1168	0.0368	−0.0427	0.1309	0.0675	0.0708	0.0699	0.0602	0.057	−0.0145	0.0706	0.05
HWE(*P*)	0.129	0.552	0.122	0.155	0.222	0.301	0.229	0.139	0.164	0.514	0.019	0.072
*C*. *gunnari (SGb)*												
*N*	96	93	88	94	96	86	94	94	95	87	92	96
*A*	12	16	7	6	5	13	22	6	18	14	11	25
*H*_o_	0.79	0.89	0.72	0.55	0.27	0.63	0.69	0.63	0.79	0.78	0.65	0.93
*H*_e_	0.8	0.87	0.72	0.56	0.3	0.72	0.71	0.71	0.83	0.83	0.67	0.91
*F*_IS_	0.0153	−0.0205	0.0005	0.0034	0.11	0.1256	0.026	0.1106	0.0464	0.0553	0.0259	−0.0204
HWE(*P*)	0.433	0.171	0.059	0.213	0.212	0.093	0.215	0.185	0.097	0.557	0.573	0.522

	Nro03	Nro11	Nro27	Nro41	Nro46	Nro50	Nro66	Nro68	Nro69	Cgu69	Cgu70	
*N. rossii* (SS)												
*N*	70	77	65	78	65	73	77	71	77	52	78
*A*	8	10	13	19	12	22	9	17	16	35	11
*H*_o_	0.51	0.64	0.51	0.91	0.75	0.9	0.58	0.85	0.71	0.9	0.67
*H*_e_	0.54	0.64	0.44	0.88	0.79	0.92	0.58	0.91	0.72	0.96	0.7
*F*_IS_	0.0555	0.0003	−0.1449	−0.0327	0.0401	0.0124	−0.007	0.076	0.0046	0.0582	0.0516
HWE(*P*)	0.306	0.598	0.912	0.890	0.760	0.417	**0.000**	0.449	0.662	0.115	0.872
*N*. *rossii* (EI)												
*N*	47	47	45	48	40	29	48	45	48	48	44
*A*	6	10	9	14	9	18	8	17	16	31	8
*H*_o_	0.43	0.64	0.38	0.9	0.62	0.93	0.75	0.82	0.73	0.92	0.73
*H*_e_	0.41	0.63	0.34	0.88	0.73	0.92	0.61	0.91	0.74	0.95	0.71
*F*_IS_	−0.0468	−0.0126	−0.1233	−0.0213	0.1435	−0.0116	−0.2396	0.0949	0.0115	0.0347	−0.0281
HWE(*P*)	1.000	0.479	1.000	0.123	0.055	0.520	0.084	0.095	0.600	0.121	0.635
*N*. *rossii* (SO)												
*N*	6	6	6	6	3	5	6	6	6	6	6
*A*	3	4	4	6	2	6	5	6	4	9	5
*H*_o_	0.33	0.83	0.67	1	0.33	0.8	0.67	1	0.5	0.67	0.83
*H*_e_	0.29	0.58	0.51	0.78	0.5	0.76	0.68	0.81	0.42	0.86	0.67
*F*_IS_	−0.1429	−0.4286	−0.2973	−0.2857	0.3333	−0.0526	0.0204	−0.2414	−0.2	0.2258	−0.25
HWE(*P*)	1.000	1.000	1.000	0.919	1.000	0.796	0.514	0.763	1.000	0.034	1.000
*N. rossii* (SG)												
*N*	79	83	75	84	85	77	69	73	76	83	60
*A*	4	11	10	15	12	20	7	21	21	40	10
*H*_o_	0.35	0.59	0.45	0.88	0.8	0.87	0.48	0.88	0.7	0.94	0.6
*H*_e_	0.36	0.67	0.44	0.86	0.75	0.92	0.61	0.92	0.74	0.97	0.67
*F*_IS_	0.0134	0.1198	−0.0282	−0.0287	−0.0735	0.0555	0.2177	0.0458	0.0518	0.0262	0.105
HWE(*P*)	0.913	0.196	0.814	0.203	0.973	0.215	**0.000**	0.571	0.356	0.157	0.357
